# Commentary: Sorbitol treatment extends lifespan and induces the osmotic stress response in *Caenorhabditis elegans*

**DOI:** 10.3389/fgene.2015.00364

**Published:** 2016-01-12

**Authors:** Alan R. Hipkiss

**Affiliations:** Aston Research Centre for Health Ageing, School of Life and Health Sciences, Aston UniversityBirmingham, UK

**Keywords:** lifespan, glycolysis, glycation, methylglyoxal, triose-phosphates, glycerolphosphate dehydrogenase

It has recently been reported in this journal that exposure of *Caenorhabditis elegans* to osmotic stress, induced by sorbitol and trehalose, provokes lifespan extension which appears to be dependent on the upregulation of the enzyme glycerol-3-phosphate dehydrogenase but seemingly independent of previously recognized regulatory agents normally closely associated with aging and lifespan regulation (sirtuin activity, insulin-like growth factor signaling, and AMP kinase function; Chandler-Brown et al., 2015). A possible metabolic explanation is suggested below.

It is well recognized that suppression of glycolytic activity can delay age-related dysfunction and extend lifespan (Ingram and Roth, 2015). Aging is frequently accompanied by macromolecular modification induced by toxic metabolites, whose generation is inhibited especially when glycolysis is decreased by procedures such as caloric restriction, intermittent fasting, 2-deoxyglucose, rapamycin-induced mTOR inhibition and insulin and insulin-like growth factor signaling dysfunction.

A major age-associated macromolecular post-synthetic modification is non-enzymatic glycosylation (glycation) brought about mostly by way of reactive bicarbonyls, of which methylglyoxal (MG) is a predominant example. MG is a highly reactive decomposition product of the glycolytic triose-phosphate intermediates, dihydroxyacetone phosphate (DHAP) and glyceraldehyde-3-phosphate (GA-3-P), which are also potent glycating agents in their own right (Allaman et al., 2015). MG is regarded as a dominant source of the secondary modifications associated with type-2 diabetes (Uchiki et al., 2012; Maessen et al., 2015), and whose generation is increased post-prandially and even more so following consumption of high glycemic index diets (Uchiki et al., 2012; Whitcomb et al., 2015). Aging is frequently accompanied by proteostatic dysfunction which, at least in part, may be due to increased MG generation resulting in glycation of ubiquitin, chaperone proteins and components of the autophagic system (Uchiki et al., 2012). Consequently, those treatments which partially suppress glycolytic flux (outlined above) will suppress glycation by decreasing formation of DHAP and GA-3-P, which in turn will decrease MG generation.

Thus it follows that, because osmotic stress induces synthesis of the osmolite glycerol, presumably from glycolytic triosephosphates via the activity of glycerol-3-phosphate dehydrogenase to form glycerol-3–phosphate (which is eventually dephosphorylated to glycerol), this will lower intracellular triose-phosphate levels, thereby decreasing the potential for MG formation and thus macromolecular glycation will be correspondingly lessened. Conversion of triosephosphate to glycerol-3-phosphate will also regenerate NAD^+^ from NADH: NAD^+^ supplementation has been shown to delay aging and extend lifespan presumably via sirtuin activation (Verdin, 2015).

The proposed mechanism explains why deletion of glycerol-3-phosphate dehydrogenase genes eliminates the lifespan-extending effects of sorbitol treatment (Chandler-Brown et al., 2015). It is also interesting to note that addition of glycerol to *C. elegans* has been shown to accelerate aging and decrease nematode lifespan (Lee et al., 2009). Thus it can concluded that it is not the presence of glycerol *per se* which exerts the beneficial effects on *C. elegans* lifespan, but the metabolic processes employed in its generation. A study of the effects osmotic stress on intracellular concentration of triose-phosphates (Deng et al., 2016) in *C. elegans* would test the veracity this proposal.

## Conflict of interest statement

The author declares that the research was conducted in the absence of any commercial or financial relationships that could be construed as a potential conflict of interest.
